# Aldehyde dehydrogenase and ATP binding cassette transporter G2 (ABCG2) functional assays isolate different populations of prostate stem cells where ABCG2 function selects for cells with increased stem cell activity

**DOI:** 10.1186/scrt343

**Published:** 2013-10-25

**Authors:** Kalyan J Gangavarapu, Gissou Azabdaftari, Carl D Morrison, Austin Miller, Barbara A Foster, Wendy J Huss

**Affiliations:** 1 Departments of Pharmacology and Therapeutics, Roswell Park Cancer Institute, Buffalo, NY, USA; 2 Department of Pathology, Roswell Park Cancer Institute, Buffalo, NY, USA; 3 Department of Biostatistics, Roswell Park Cancer Institute, Buffalo, NY, USA; 4 Department of Urologic Oncology, Roswell Park Cancer Institute, Buffalo, NY, USA

## Abstract

**Introduction:**

High expression of aldehyde dehydrogenase1A1 (ALDH1A1) is observed in many organs and tumors and may identify benign and cancer stem cell populations.

**Methods:**

In the current study, the stem cell characteristics were determined in cells isolated from human prostate cell lines and clinical prostate specimens based upon the ALDEFLUOR™ assay. Cells isolated based on the ALDEFLUOR™ assay were compared to cells isolated based on ATP binding cassette transporter G2 (ABCG2) activity using the side population assay. To test for stem cell characteristics of self-renewal and multipotency, cells with high and low ALDH1A1 activity, based on the ALDEFLUOR™ assay (ALDH^Hi^ and ALDH^Low^), were isolated from prostate clinical specimens and were recombined with rat urogenital sinus mesenchyme to induce prostate gland formation.

**Results:**

The percentage of ALDH^Hi^ cells in prostate cell lines (RWPE-1, RWPE-2, CWR-R1, and DU-145) was 0.5 to 6%, similarly in non-tumor and tumor clinical specimens the percentage of ALDH^Hi^ cells was 0.6 to 4%. Recombinants using ALDH^Hi^ cells serially generated prostate tissue up to three generations with as few as 250 starting cells. Immunohistochemical analysis of the recombinants using ALDH^Hi^ cells contained prostatic glands frequently expressing androgen receptor (AR), p63, chromogranin A, ALDH1A1, ABCG2, and prostate specific antigen (PSA), compared to their ALDH^Low^ counterparts. Inhibition of ALDH resulted in the reduction of sphere formation capabilities in the CWR-R1, but not in the RWPE-2 and DU-145, prostate cell lines. ABCG2 inhibition resulted in a more robust decrease of sphere formation in androgen sensitive cell lines, CWR-R1 and RWPE-2, but not androgen insensitive DU-145. *ALDH1A1* expression was enriched in ALDH^Hi^ cells and non-side population cells. *ABCG2* expression was only enriched in side population cells.

**Conclusions:**

The percentage of ALDH^Hi^ cells in prostate cell lines and prostate tissue was consistently higher compared to cells with high ABCG2 activity, identified with the side population assay. The expression of the stem and differentiation markers indicates the ALDH^Hi^ recombinants contained cells with self-renewal and multipotency activity. When the two assays were directly compared, cells with the side population phenotype demonstrated more stem cell potential in the tissue recombination assay compared to ALDH^Hi^ cells. The increased stem cell potential of side population cells in the tissue recombination assay and the decrease in sphere formation when ABCG2 is inhibited indicates that the side population enriches for prostate stem cells.

## Introduction

Cytoprotective activity is proposed to protect stem cells from mutations that can arise from cytotoxic insults. Therefore, stem cells are often isolated based on cytoprotective activity. Conversely, stem cell assays have been developed exploiting a cytoprotective mechanism. Two examples of assays based on stem cell cytoprotective activity are: (1) the side population assay based on the efflux of Hoechst 33342 fluorescent dye by the ATP-binding cassette (ABC) transporters [1] and; (2) high activity of aldehyde dehydrogenases (ALDH) detoxifying enzymes [2]. The ALDEFLUOR™ assay was developed based on the second property [2]. ABC transporters remove cytotoxins and regulatory signals (Reviewed in [3]). ALDHs catalyze the irreversible oxidation of several different endogenously and exogenously produced aldehydes into the corresponding carboxylic acids (Reviewed in [4]).

The human ALDH superfamily consists of 19 members where ALDH1A1 and ALDH3A1 are thought to be important in stem cell protection, differentiation and expansion [2,4-7]. Members of the ALDH family have been identified as markers for both normal and cancer stem cells in different tissues [4-7]. Specifically, ALDH1A1 and ALDH3A1 have been used as markers to isolate normal and cancer stem cells and have a potential functional role in normal and cancer stem cells (Reviewed in [4]). Van den Hoogen *et al*. determined expression of several ALDH enzyme isoforms in primary prostate tumors and primary prostate cultures and expression of ALDH1A1, ALDH4A1, ALDH7A1 and ALDH9A1 were elevated [8]. ALDH activity is emerging as a promising marker that can be used to identify and isolate normal and cancer stem cells [9].

The ALDEFLUOR™ assay is based on the conversion of Bodipy aminoacetaldehyde (BAAA), an ALDH substrate, to highly fluorescent Bodipy aminoacetate (BAA). Thus, cells with high ALDH1A1 activity (ALDH^Hi^) can be isolated using fluorescence activated cell sorting (FACS) [2]. High levels of ALDH expression and activity in stem cells from various cancers including bladder, breast, lung, ovarian, liver, colon, pancreas and prostate have been detected using the ALDEFLUOR™ assay [10-18]. High ALDH1A1 activity is associated with poor prognosis in patients with breast and prostate cancer [18,19]. Li *et al*. found that high ALDH1A1 expression in clinical prostate specimens correlated with poor prostate cancer patient survival and Gleason Score [18]. The studies also tested the tumorigenicity of ALDH^Hi^ and ALDH^Low^ cells isolated from LNCaP and PC-3 prostate cancer cell lines. A minimum of 500 ALDH^Hi^ cells from LNCaP and PC-3 prostate cancer cell lines generated serially transplantable prostate tumors containing neuroendocrine and secretory cells, suggesting multipotentiality [18]. In separate studies, as few as 100 ALDH^Hi^ cells from the PC-3 M cell line had tumor forming and metastatic ability [8]. ALDH^Hi^ cells isolated from epithelial ovarian cancer cell lines are shown to have more sphere-forming capabilities, are more tumorigenic, and contribute to chemotherapy resistance as compared to ALDH^Low^ cells. This suggests that ALDH^Hi^ cells are enriched for cancer stem-like cells [20]. The inhibition of ALDH1, with diethylaminobenzaldehyde (DEAB), reduced cancer stem cell characteristics of chemotherapy and radiation resistance in ALDH^Hi^ breast cancer cells [21].

In cell lines from different cancer types, ALDH^Hi^ cells have higher sphere formation ability, tumorigenicity and are more chemo- and radio-resistant compared to ALDH^Low^ cells. However, ALDH^Hi^ cells have not been isolated from clinical specimens of prostate cancer, or tested in prostate stem cell assays. In the current study, we tested ALDH^Hi^ cells isolated from cell lines and human prostate specimens for stem cell properties. ALDH^Hi^ and ALDH^Low^ cells from clinical specimens were evaluated for stem cell characteristics using tissue recombination with rat urogenital mesenchyme (rUGM) for serial prostate regenerative capability compared to cells isolated based on the side population assay. The requirement for ALDH and ABCG2 activity was determined in the sphere formation assay.

## Methods

### Ethics statement

All of the tissue samples were collected under an Institutional Review Board (IRB)-approved exempt non-human research protocol at Roswell Park Cancer Institute (RPCI). Specimens were collected after IRB-approved written consent from the patient was obtained at RPCI. All experiments were conducted and approved under our Institutional Animal Care and Use Committee (IACUC) at RPCI under protocol ID number 1201 M.

### Cell lines and culture

Prostate cell lines RWPE-1 American Type Culture Collection (ATCC, Manassas, VA, USA), RWPE-2 (ATCC), CWR-R1 and DU-145 were maintained in culture medium as described previously [22]. Briefly, RWPE-1 and RWPE-2 cells were maintained in Keratinocyte Serum Free Medium supplemented with bovine pituitary extract and epidermal growth factor. DU-145 cells were maintained in Roswell Park Memorial Institute (RPMI) 1640 media supplemented with 10% FBS, L-glutamine, penicillin and streptomycin. CWR-R1 cells were maintained in Richter’s improved MEM supplemented with 2% FBS, HEPES, ITS, epidermal growth factor, linoleic acid and nicotinamide. All cell lines were cultured at 37°C in an atmosphere of 5% CO_2_. Cells were grown to 90% confluence and harvested by incubation with trypsin-EDTA.

### Human prostate clinical specimen digestion

Fresh human benign prostate tissue and prostate cancer tissue (>90% cancer), identified by analysis of frozen sections from radical prostatectomy surgical specimens, was obtained from the Pathology Resource Network at RPCI as described previously [23]. All prostate tissue was obtained in accordance with National Institutes of Health (NIH) guidelines on the use of human subjects and with the approval of the RPCI Internal Research Board. Enzymatic tissue digestion with dispase and single cell suspension preparation was performed as described previously [22,24].

### Gating strategy used for FACS isolation of ALDH^Hi^ and ALDH^Low^ cells

Cells isolated from prostate tissue were stained with the ALDEFLUOR™ reagent from the ALDEFLUOR™ assay kit (Stem Cell Technologies, Vancouver, BC, Canada) according to the manufacturer’s protocol and as previously described [2,13]. The assay was optimized to interact with human ALDH1A1 according to the manufacturer’s protocol and as previously described [2,13]. Briefly, the isolated cells were stained with 1 μM ALDEFLUOR™ reagent and incubated at 37°C for 45 to 60 minutes. RWPE-1 and RWPE-2 prostate cells were stained with 0.1 μM ALDEFLUOR™ reagent. CWR-R1 and DU-145 prostate cells were stained with 0.5 μM ALDEFLUOR™ reagent. After incubation, the cells were centrifuged at 950 × g and resuspended in 0.5 ml ALDEFLUOR™ assay buffer. The cells were labeled with 7-aminoactinomycin D (7-AAD) at a concentration of 5 μl/500 μl to eliminate dead cells. Cells were sorted into ALDH^Hi^ and ALDH^Low^ populations with a BD FACS Aria II cell sorter (BD Bioscience, San Jose, CA, USA). Cells with high fluorescence indicate high ALDH1A1 activity, ALDH^Hi^ cells, were detected and the dead cells were eliminated using 7-AAD. DEAB (15 μM), a potent inhibitor of ALDH1 activity, was added to establish the ALDH^Hi^ population. Forward scatter and side scatter plots were used to reduce gating doublets and obtain a singlet population.

### Gating strategy used for FACS isolation of side population and non-side population cells

Cells isolated from prostate tissue were stained with 10 μM Vybrant® DyeCycle™ Violet (DCV) (Invitrogen, Carlsbad, CA, USA) according to a protocol modified from Telford *et al*. [25] and 7-AAD as previously described [22,26]. The gating strategy for sorting the side and non-side populations was performed as previously described [22,26]. Viable cells were sorted based upon DCV content into side and non-side populations with a BD FACS Aria II cell sorter.

### Sphere formation assay

The sphere formation assay was performed with RWPE-2, CWR-R1 and DU-145 prostate cells by treatment in the presence of 5, 10 and 20 μM DEAB or 1 and 5 μM Ko143. DEAB was prepared in 95% EtOH, and Ko143 was prepared in DMSO. ETOH (95% EtOH) or DMSO treatment was used as the vehicle control. Cells were plated at a density of 5,000 cells/well in ultra-low attachment 24-well plates (VWR-Cat # 29443–032, Radnor, PA, USA) in quadruplicate, and the experiment was performed two times. Cells were suspended in 40 μL medium and mixed thoroughly with 60 μL BD Matrigel™ (BD Bioscience, San Jose, CA, USA, Cat # 354234,). The mixture was pipetted around the rim of the well, and the plate was incubated in a 5% CO_2_ incubator at 37°C for 45 minutes to allow the BD Matrigel™ to solidify. A total of 900 μL of appropriate media was added to each well and plates were maintained in 5% CO_2_ incubator at 37°C for 10 to 14 days. Spheres in each well were quantitated after 10 to 14 days visually under a microscope at 10× magnification.

### RNA isolation and cDNA synthesis

RNA isolated from the side population, non-side population, ALDH^Hi^ and ALDH^Low^ cells CWR-R1 prostate cancer cells was used to determine the expression of *ABCG2* and *ALDH1* genes. RNA was isolated using RNAeasy micro kit (Qiagen, Valencia, CA, USA, Cat # 74004) according to the manufacturer’s protocol, and the concentration of total RNA was determined using the NanoDrop 8000 spectrophotometer (Wilmington, DE, USA). Total RNA (50 ng) from each sample was reverse transcribed into single-stranded cDNA with a SuperScript III First-Strand cDNA synthesis kit (Invitrogen, Carlsbad, CA, USA, Cat, # 18080–051) using oligo (dT) primers, according to the manufacturer’s protocol.

### Real-time PCR

Real-time PCR was performed to determine gene expression. The synthesized cDNA was amplified by real-time PCR using a SYBR Green master mix kit (Applied Biosystems, Carlsbad, CA, USA, Cat # 4309155). The reactions were carried out in a total volume of 20 μL. Analyses for *ABCG2, ALDH1A1, ALDH4A1, ALDH7A1, ALDH9A1* and *GAPDH* were performed. Conditions for the PCR were: 50°C for 2 minutes, 95°C for 10 minutes, followed by 40 cycles each run at 95°C for 15 seconds and 60°C for 1 minute. The primers (Integrated DNA Technologies, Coralville, IA, USA) used were: *ABCG2* (0.25 μM) sense, 5′ – AGCAGGATAAGCCACTCATAGA – 3′;

*ABCG2* (0.25 μM) antisense, 5′ – GTTGGTCGTCAGGAAGAAGAG – 3′;

*ALDH1A1* (0.5 μM) sense, 5′ – TTACCTGTCCTACTCACCGATT – 3′;

*ALDH1A1* (0.5 μM) antisense, 5′ – GCCTTGTCAACATCCTCCTTAT – 3′;

*ALDH4A1* (0.5 μM) sense, 5′ – GGACGTGCAGTACCAAGTGT – 3′;

*ALDH4A1* (0.5 μM) antisense, 5′ – TAGGCTTCAGGTCCCACTCT – 3′;

*ALDH7A1* (0.5 μM) sense, 5′ – CAACGAGCCAATAGCAAGAG – 3′;

*ALDH7A1* (0.5 μM) antisense, 5′ – GCATCGCCAATCTGTCTTAC – 3′;

*ALDH9A1* (0.5 μM) sense, 5′ – GCTGCCAGGATAATAAGGGA – 3′;

*ALDH9A1* (0.5 μM) antisense, 5′ – GAAATGTCAATGTCCAAGCG – 3′;

*GAPDH* (0.2 μM) sense, 5′ – GAACATCATCCCTGCCTCTACT – 3′;

*GAPDH* (0.2 μM) antisense, 5′ – CGCCTGCTTCACCACCTT -3′.

Reactions were run and analyzed using an Applied Biosystems 7300 fast real-time PCR system (Carlsbad, CA, USA). Gene expression was normalized to *GAPDH* and fold change in gene expression was calculated using the following formula: fold change in gene expression = 2^-(ΔΔdCt)^.

### Tissue recombinant technique

Rat urogenital sinus mesenchyme was dissected as described previously [27,28]. First generation recombinants were made by aliquoting 50 to 2,000 ALDH^Hi^ or ALDH^Low^ cells or 50 to 250 side population or non-side population cells isolated from human prostate tissue specimens with a single rUGM in a 200 μL microcentrifuge tube. Sorted cells were spun onto the rUGM, or in a separate tube sorted cells were pelleted alone as a negative control. Sorted cells + rUGM and sorted cell pellets were suspended in collagen as described previously [26].

### Renal grafting

Cells plus rUGM or cells alone in collagen were grafted under the renal capsule in 12-week-old SCID male CbySmn. CB 17 Prkdcscid mice, which were castrated and implanted subcutaneously with 12.5 mg sustained-release testosterone pellets (Innovative Research of America, Sarasota, FL, USA) as described previously [26]. Following 6 to 12 weeks of growth, grafts were harvested and weighed. When ductal growth was present, grafts were micro-dissected and a portion was used for serial recombination with new rUGM as described previously [26]. Briefly, an approximately 1 mm cube of tissue containing both epithelial and stromal compartments was micro-dissected from the graft and used for serial recombination with new rUGM [26]. The remaining portion of the recombinant was fixed in neutral buffered formalin as described previously [26].

### Identification of the origin of prostate generated using fluorescence *in situ* hybridization (FISH)

In order to identify the cells contributing to the prostate epithelium in the tissue recombinants FISH was performed (Additional file 1: Figure S1). Mammalian telomeres were detected as described previously [26]. The number of human prostatic glands in each recombinant was determined as previously described [26].

### Immunohistochemistry (IHC)

Immunohistochemical analysis was performed to examine the expression of prostate differentiation markers as described previously [26]. ALDH1A1 was detected using a 1:80 dilution of rabbit monoclonal anti-ALDH1A1 (cat # NB110-55451, Novus Biologicals, Littleton, CO, USA). Human prostate tissue served as a positive control, and no primary antibody controls served as negative controls in all experiments (Additional file 2: Figure S2).

### Aperio imaging recombinant histology and IHC

All slides containing stained recombinants were scanned on the ScanScope XT System in the Pathology Resource Network (Buffalo, NY, USA) at RPCI, and images were analyzed with Spectrum Version 10.2.2.2317 (Vista, CA, USA).

### Statistical analysis

Fisher’s exact test, Student’s *t*-test and One-way ANOVA were performed with GraphPad Prism version 6.00 software (La Jolla, CA, USA). The recombinant survival rate was defined as the proportion of recombinants demonstrating growth at each generation (cycle) analyzed. Recombinants with growth in the third generation were censored. The Kaplan-Meier analysis performed using SAS v9.3 software (Cary, NC, USA) provides visual comparison of these survival distributions for the ALDH^Hi^ and ALDH^Low^ recombinants. The null hypothesis of no difference in the survival distributions was assessed using the Logrank test.

## Results

### Isolation of ALDH^Hi^ and ALDH^Low^ cells from prostate cell lines

**Figure 1:**
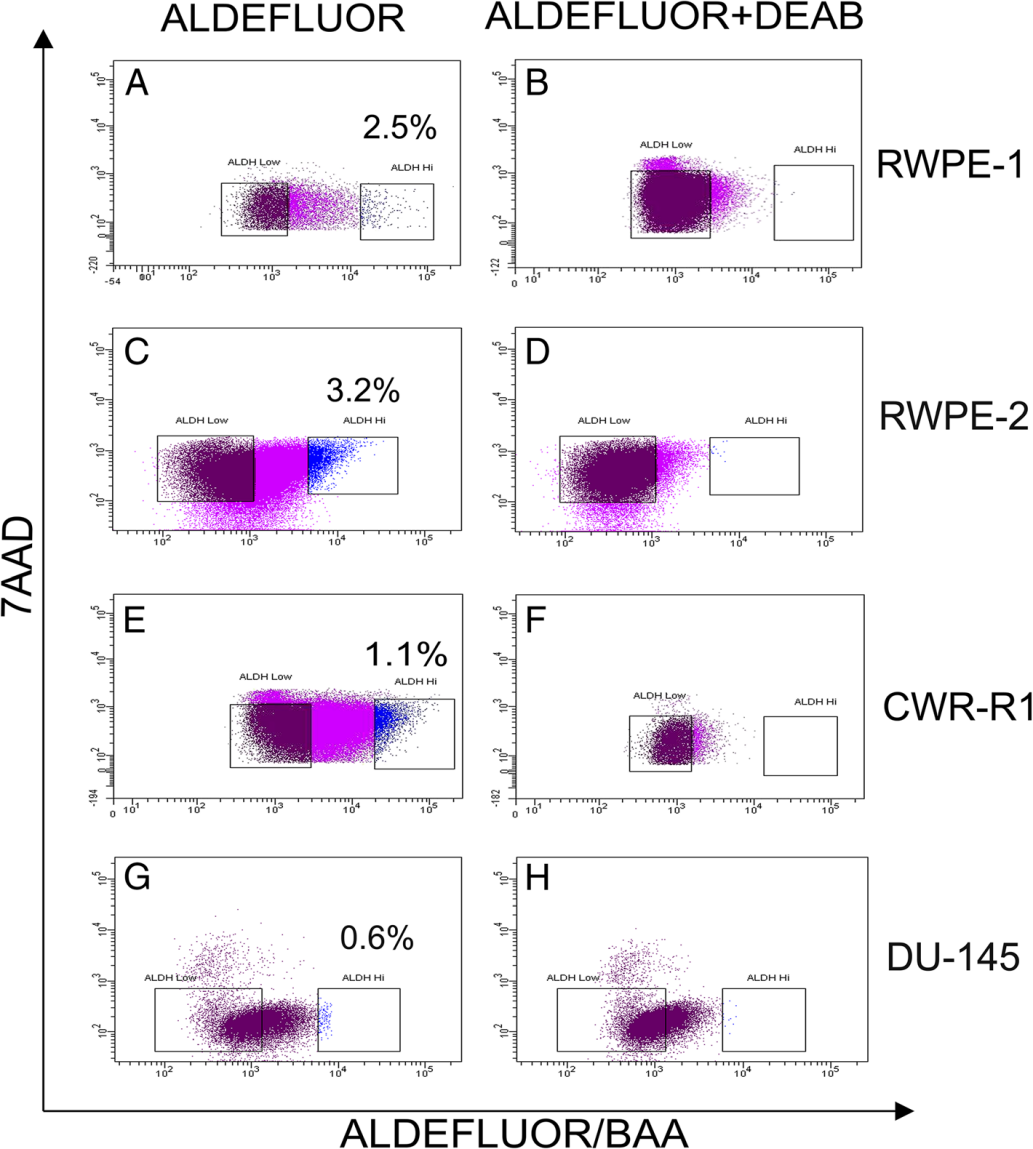
**Isolation of ALDH**^**Hi **^**and ALDH**^**Low **^**cells from prostate cells by ALDEFLUOR™ assay.** RWPE-1, RWPE-2, CWR-R1 and DU-145 prostate cells were stained with ALDEFLUOR™ reagent and analyzed using BD FACS Aria II sorter. ALDH^Hi^ cells were detected in the higher fluorescent region **(A, C, E** and **G)**. ALDH^Hi^ population was inhibited with DEAB resulting in no ALDH^Hi^ cell detection **(B, D, F** and **H)**. The ALDH^Low^ population is gated with a clear separation from the ALDH^Hi^ population.

The ALDEFLUOR™ assay was performed on RWPE-1 RWPE-2, CWR-R1 and DU-145 prostate cell lines. ALDH^Hi^ cells represented 0.5 to 6% of the total cell population in RWPE-1, RWPE-2, CWR-R1 and DU-145 prostate cells (Figures 1A, C, E, G). ALDH^Hi^ cells are gated based upon the absence of the population in the presence of DEAB, an inhibitor of ALDH1, (Figures 1B, D, F, H) and the ALDH^Low^ cells were gated with a clear separation from the ALDH^Hi^ cells (Figures 1A, C, E, G).

### Prostate generation in recombinants with ALDH^Hi^ and ALDH^Low^ cells isolated from human prostate specimens

**Figure 2:**
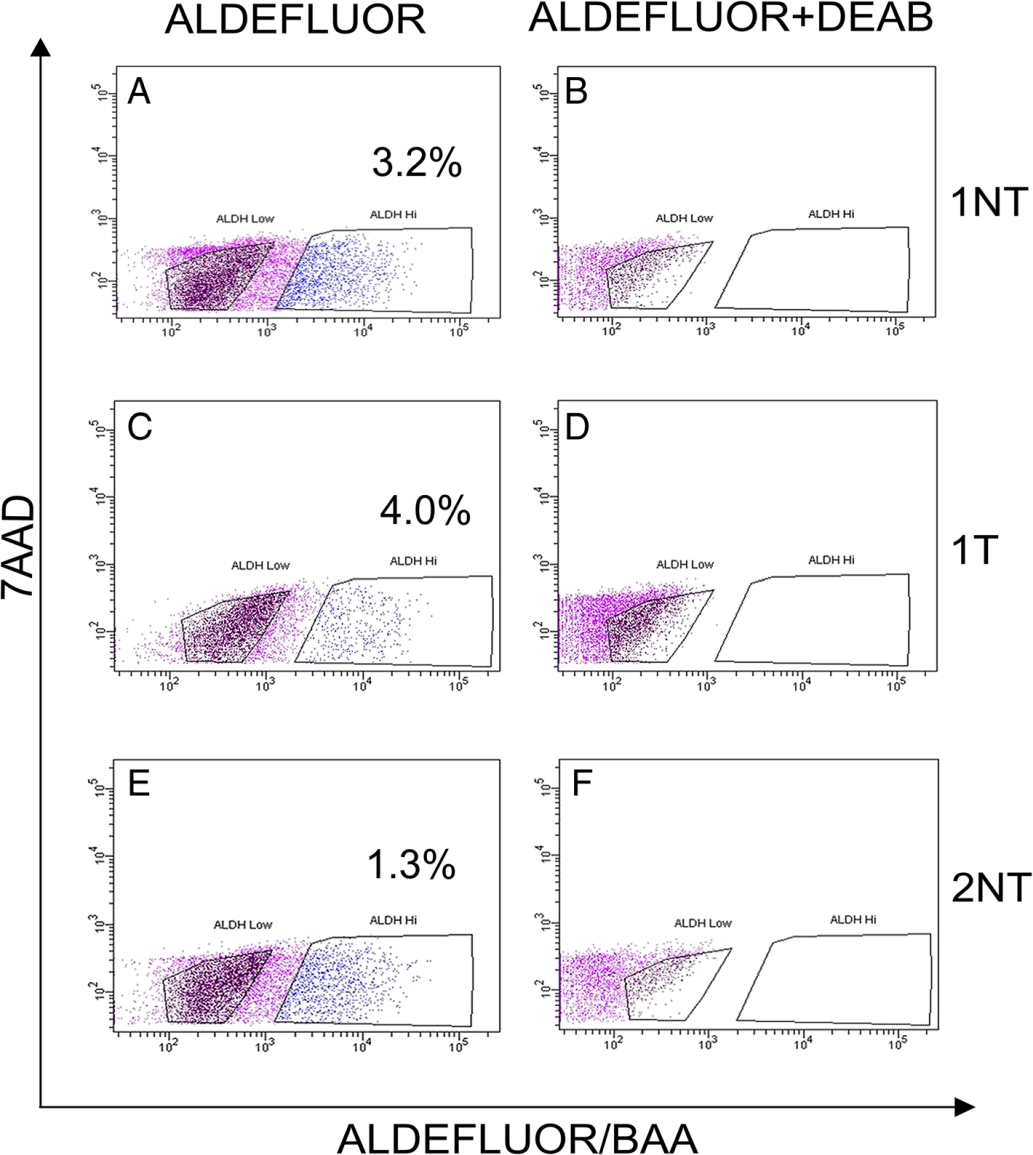
**Isolation of ALDH**^**Hi **^**and ALDH**^**Low **^**cells from clinical human prostate specimen by ALDEFLUOR™ assay.** Cells isolated from clinical human prostate specimen were stained with ALDEFLUOR™ reagent and analyzed using BD FACS Aria II sorter. ALDH^Hi^ cells were detected in the higher fluorescent region **(A, C** and **E)**. ALDH^Hi^ population was inhibited with DEAB resulting in no ALDH^Hi^ cell detection **(B, D** and **F)**. ALDH^Low^ population is gated with a clear separation from the ALDH^Hi^ population.

The ALDEFLUOR™ assay was performed on six radical prostatectomy specimens. ALDH^Hi^ cells represented 0.6 to 4% of the cells in non-tumor, tumor and mixed prostate specimens (Figure 2 and Table 1). ALDH^Hi^ or ALDH^Low^ cells were recombined with rUGMs in the tissue recombinant assay in order to functionally test for stem cell properties. Sorted cells suspended in collagen without rUGM were used as a non-inductive control to test tumorigenic capacity. Equal numbers of cells (50 to 2,000) were used for each sorted population (Table 2). At the end of each grafting period, the grafts were harvested, weighed and examined microscopically, blinded to the contributing population, for glandular formation. Recombinants that demonstrated ductal growth were micro-dissected, and a small portion (approximately 1 mm^3^) of the ductal structure was recombined with new rUGM and implanted under the renal capsule of host mice. The remaining portion of each recombinant was formalin-fixed and paraffin-embedded for IHC analysis. If under the dissecting microscope there was no evidence of ductal growth or the ductal growth was obviously rodent, the recombinant was processed for histological analysis of microscopic ductal growth, and no serial recombination with rUGM was performed. This procedure resulted in some recombinants with histological growth not being serially passed and some recombinants being serially passed, although there was no histological evidence of human epithelial cell growth.

**Table 1:** **Viable ALDH**^**Hi**^**, ALDH**^**Low**^**, side population and non-side population cells in human prostate tissue specimens**

**Specimen**	**Gleason grade**	**Specimen weight (g)**	**# Viable cells**	**% Viable cells**	**% ALDH**^**Hi**^	**% ALDH**^**Low**^
1NT	3 + 4	2.994	3.31 × 10^5^	22.3	3.2	13.7
1 T	3 + 4	1.451	6.47 × 10^5^	26.6	4.0	14.9
2NT	3 + 3	6.105	1.03 × 10^6^	15.8	1.3	11.2
3NT	4 + 3	1.678	1.78 × 10^5^	10.5	1.0	2.2
4 M	4 + 5	1.540	0.55 × 10^5^	22.4	1.1	2.1
4 T		5.387	1.57 × 10^5^	36.5	0.6	8.2
5NT	4 + 3	3.314	1.18 × 10^5^	33.6	1.0	1.4
6 T	5 + 4	0.977	1.84 × 10^5^	27.5	1.0	22.2
					**%SP**	**%NSP**
5NT	4 + 3	3.314	0.81 × 10^5^	59.5	0.3	16.7
6 T	5 + 4	0.977	0.66 × 10^5^	11.0	0.3	2.1

M, Mixed prostate specimen from radical prostatectomies; NSP, Non-Side Population; NT, Histologically non-tumor areas; SP, Side Population; T, Histologically tumor areas. Percentage, percent of sorted populations was calculated based upon total viable cells from each specimen.

**Table 2:** **Incidence of ductal growth in ALDH**^**Hi **^**and ALDH**^**Low **^**recombinants**

**Prostate Ductal Growth**^**(#Analyzed)**^**[Weeks Grafted]**							
		**ALDH**^**Hi **^**Generation**			**ALDH**^**Low **^**Generation**			
**Specimen**	**# of Cells**	**1**^**st**^	**2**^**nd**^	**3**^**rd**^	**1**^**st**^	**2**^**nd**^	**3**^**rd**^	
1NT	250	0^(3)^[7]			1^(3)^[7]	1^(1)^[10]	
1NT	1,000	1^(3)^[7]	1^(1)^[10]	1^(1)^[9]	1^(3)^[7]	1^(3)^[10]	
1T	250	1^(3)^[7]	1^(1)^[10]	1^(1)^[9]	0^(3)^[7]		
1T	1,000	2^(3)^[7]	2^(2)^[10]		0^(1)^[7]		
2NT	2,000	2^(3)^[7]	2^(2)^[10]	1^(1)^[9]	1^(2)^[7]	1^(1)^[10]	
3NT	500	1^(3)^[10]	1^(1)^[9]		2^(3)^[10]	1^(2)^[9]	
3NT	2,000	1^(3)^[10]	0^(1)^[9]		1^(3)^[10]	1^(1)^[9]	
4M	250	0^(3)^[10]			2^(3)^[7]	0^(2)^[8]	
4M	1,000	2^(3)^[7]	0^(2)^[8]		0^(3)^[7]		
4T	50	0^(3)^[7]			1^(3)^[7]		
4T	250	0^(3)^[7]			0^(3)^[7]		
5NT	50	0^(3)^[12]			0^(3)^[12]		
5NT	250	1^(2)^[12]	1^(1)^[12]		1^(2)^[12]	1^(1)^[12]	
6T	250	1^(2)^[8]	0^(1)^[6]		0^(3)^[8]		
6T	500	2^(2)^[8]			0^(3)^[8]		
**Total**		**14**^**(42)**^	**8**^**(12)**^	**3**^**(3)**^	**10**^**(41)**^	**6**^**(11)**^	

NT: Histologically non-tumor areas; T: Histologically tumor areas; M: Mixed prostate specimen from radical prostatectomies.

### Survival rate and number of human prostate glands generated by recombinants

**Figure 3:**
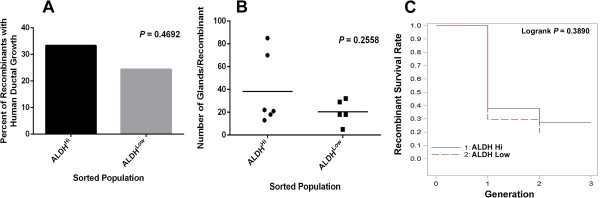
**Number of human prostate glands generated per recombinant and recombinant survival rate of recombinants grafted. A**. The percentage of recombinants with human ductal growth in the first generation ALDH^Hi^ n = 42 and ALDH^Low^ n = 41, analyzed by Fisher’s Exact test *P* = 0.4692. **B**. The number of glands per recombinant in the first generation derived from ALDH^Hi^ cells (n = 6) and ALDH^Low^ (n = 5) was quantitated and analyzed by unpaired *t*-test analysis *P* = 0.2558. **C**. Kaplan-Meier plot of recombinant survival rate in multiple generations, Logrank test *P* = 0.3890.

All recombinants were stained with H&E to evaluate prostatic gland structure. Rodent telomere repeats were used to identify rodent epithelial cell contribution to the prostatic glands in the recombinants. Each recombinant was examined for expression of rodent telomere repeat with FISH analysis (Additional file 1: Figure S1). Prostate glands that were negative for rodent telomeres were considered to be of human origin [29]. First generation growth was analyzed in 83 recombinants, in the first generation 14/42 (approximately 33%) ALDH^Hi^ recombinants and 10/41 (approximately 24%) ALDH^Low^ recombinants contained human epithelial ductal growth, but the difference was not statistically significant (Table 2, Figure 3A). All recombinant growth was evaluated by a genitourinary pathologist (GA) and was considered benign. There was no evidence of prostate cancer pathology in any of the specimens regardless of the pathology of the initial tissue used. All recombinants were stained with alpha-methylacyl-CoA racemace (AMACR), a marker of prostate cancer and prostatic intraepithelial neoplasia and no recombinants had detectable expression of AMACR (data not shown). The total number of human prostatic glands in each first generation recombinant was determined (Figure 3B), but there was no significant difference in the average number of human prostatic glands in recombinants generated with ALDH^Hi^ cells compared to ALDH^Low^ cells.

The number of ALDH^Low^ sorted cells used in tissue recombination or the length of engraftment did not seem to impact the frequency of ductal growth in the recombinants (Table 3). However, when 500 to 2,000 ALDH^Hi^ cells were used in recombination, the ductal growth rate increased to 50 to 60% compared to 0% to 19% when 50 to 250 ALDH^Hi^ cells were used (Table 3). Recombinants with a minimum of 250 ALDH^Hi^ cells serially generated prostate up to three generations, whereas no recombinant with ALDH^Low^ cells generated prostate ductal growth in the third generation (Table 3). The frequency of recombinant growth in the second and third generations was calculated as a percentage of the initial number of recombinants grafted in the first generation (Table 3).

**Table 3:** **Human ductal growth in each generation of ALDH**^**Hi **^**and ALDH**^**Low **^**recombinants**

**Number of recombinants with ductal growth (%)**								
**# of cells**	**N = # of grafts**	**ALDH**^**Hi **^**generation**			**N = # of grafts**	**ALDH**^**Low **^**generation**		
		**1**^**st**^	**2**^**nd**^	**3**^**rd**^		**1**^**st**^	**2**^**nd**^	**3**^**rd**^
50	6	0			6	1 (16.6)	0	
250	16	3 (18.7)	2 (12.5)	1 (6.2)	17	4 (23.5)	2 (11.8)	0
500	5	3 (60.0)	1 (20.0)	0	6	2 (33.3)	1 (16.6)	0
1,000	9	5 (55.5)	3 (33.3)	1 (11.1)	7	1 (14.3)	1 (14.3)	0
2,000	6	3 (50.0)	2 (33.3)	1 (16.6)	5	2 (40.0)	2 (40.0)	0
**Total**	**42**	**14 (33.3)**	**8 (19.0)**	**3 (7.1)**	**41**	**10 (24.4)**	**6 (14.6)**	**0**

The ability of the ALDH^Hi^ and ALDH^Low^ cells to generate prostatic tissue in serial generations was compared using Kaplan-Meier methods and was not statistically significant *P* = 0.389 (Figure 3C). When recombinants were harvested there was no definitive way to determine the species of origin of the ductal growth in the recombinants. Therefore, serial passage of recombinants was only performed when non-rodent ductal growth was evident upon micro-dissection. This resulted in instances when recombinants that demonstrated histological human epithelial growth were not recombined and other occasions where the ductal growth that was serially passaged did not contain human epithelium upon histological and FISH analysis. These recombinants were removed from analysis if human ductal growth was not detected in serial generations. If human ductal growth was detected in the second and/or third generation recombinants, human ductal growth was assumed in previous generations even if not histologically detected in the remaining portion not used for serial recombination.

### ALDH^Hi^ and ALDH^Low^ recombinants grafted under the renal capsule of SCID mice generated differentiated human prostate glands

**Figure 4:**
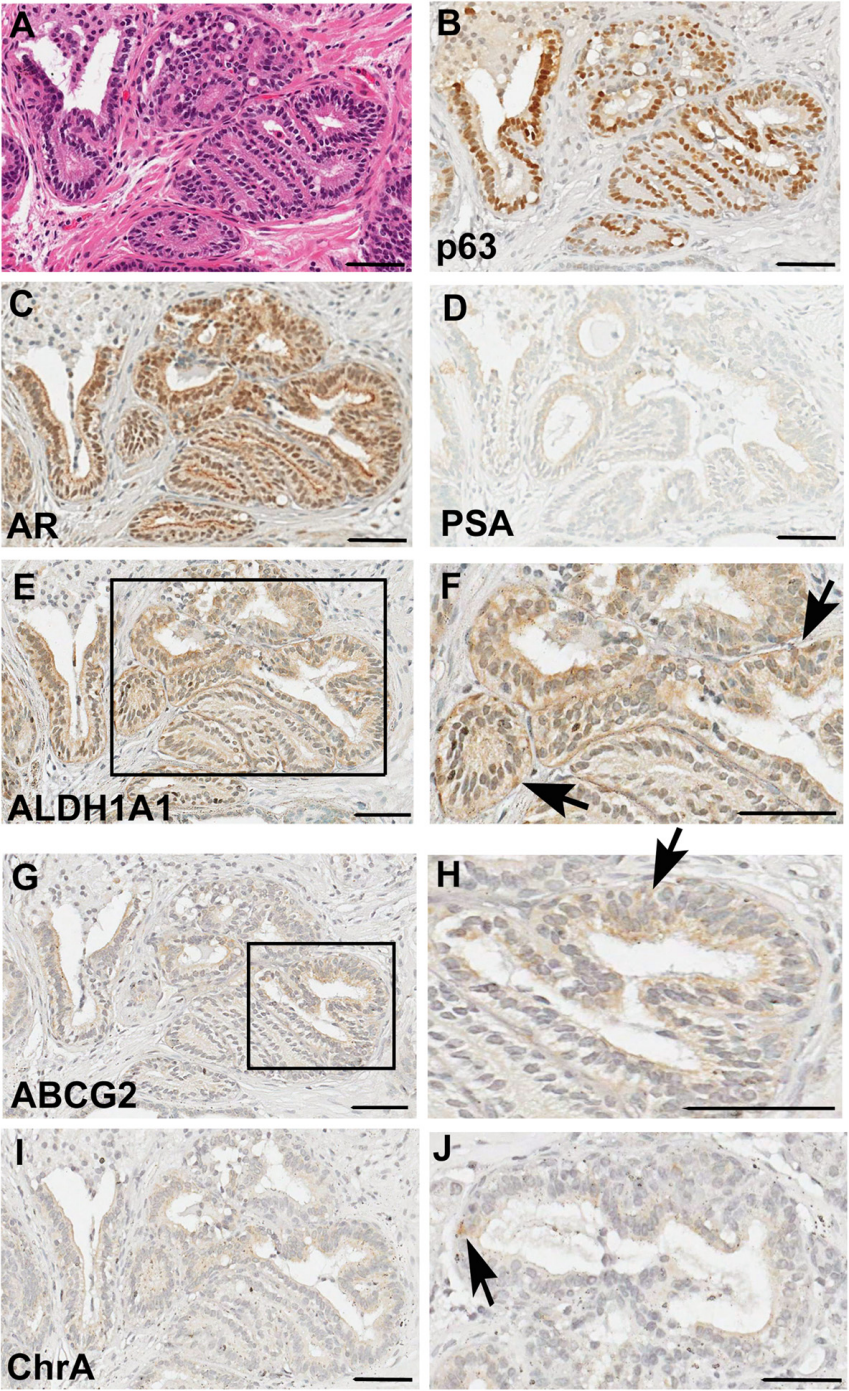
**IHC analysis of prostate differentiation markers in a recombinant using 2,000 ALDH**^**Hi **^**cells.** IHC analysis of prostate differentiation markers in a recombinant using 2,000 ALDH^Hi^ cells: **A**. H & E staining; **B**. p63 IHC; **C**. AR IHC; **D**. PSA IHC; **E**. ALDH1A1 IHC; **F**. Higher magnification of boxed area of ALDH1A1 IHC in E; **G**. ABCG2 IHC; **H**. Higher magnification of boxed area of ABCG2 IHC in G; **I**. Chromogranin A (Chr A) IHC; **J**. Higher magnification of Chromogranin A IHC in a different region of same recombinant. Arrow indicates positive cell. Scale bar = 50 μm.

All recombinants with human prostatic growth were analyzed for prostate differentiation markers. Prostate differentiation was tested by examining expression of p63, AR, PSA, ALDH1A1, ABCG2 and chromogranin A. Most glands contained cells expressing p63 and AR (Figure 4B, C). Recombinants generated from ALDH^Hi^ cells were more likely to contain cells also expressing ALDH1A1, PSA, ABCG2 or chromogranin A (Table 4). A recombinant generated with 2,000 ALDH^Hi^ cells from 3 NT (Table 1) had cells positive for all markers examined (Figure 4). High ALDH1A1 expression was observed in few recombinants in isolated cells outside the p63 expressing basal layer (Figure 4F).

**Table 4:** **Expression frequency of human prostate differentiation and stem cell markers in ALDH**^**Hi **^**and ALDH**^**Low **^**recombinants**

**Stain**	**ALDH**^**Hi **^**1**^**st **^**Generation Recombinants**	**ALDH**^**Low **^**1**^**st **^**Generation Recombinants**
ALDH1A1	8/42 (19%)	1/41 (2.4%)
PSA	14/42 (33.3%)	6/41 (14.6%)
ABCG2	19/42 (45.2%)	12/41 (29.2%)
Chromogranin A	3/42 (7.1%)	1/41 (2.4%)
p63	42/42 (100%)	41/41 (100%)
AR	42/42 (100%)	41/41 (100%)

Nineteen percent of the ALDH^Hi^ recombinants contained ALDH1A1 expressing cells within prostatic glandular structure compared to only approximately 2% of ALDH^Low^ recombinants (Table 4). Thus, results from tissue recombinant experiments showed that ALDH^Hi^ cells serially generated prostate tissue, and ALDH1A1 expressing cells are retained in the prostatic growth providing evidence of self-renewal.

### Inhibition of ALDH and ABCG2 activity decreases the sphere-forming capability of prostate cancer cells

**Figure 5:**
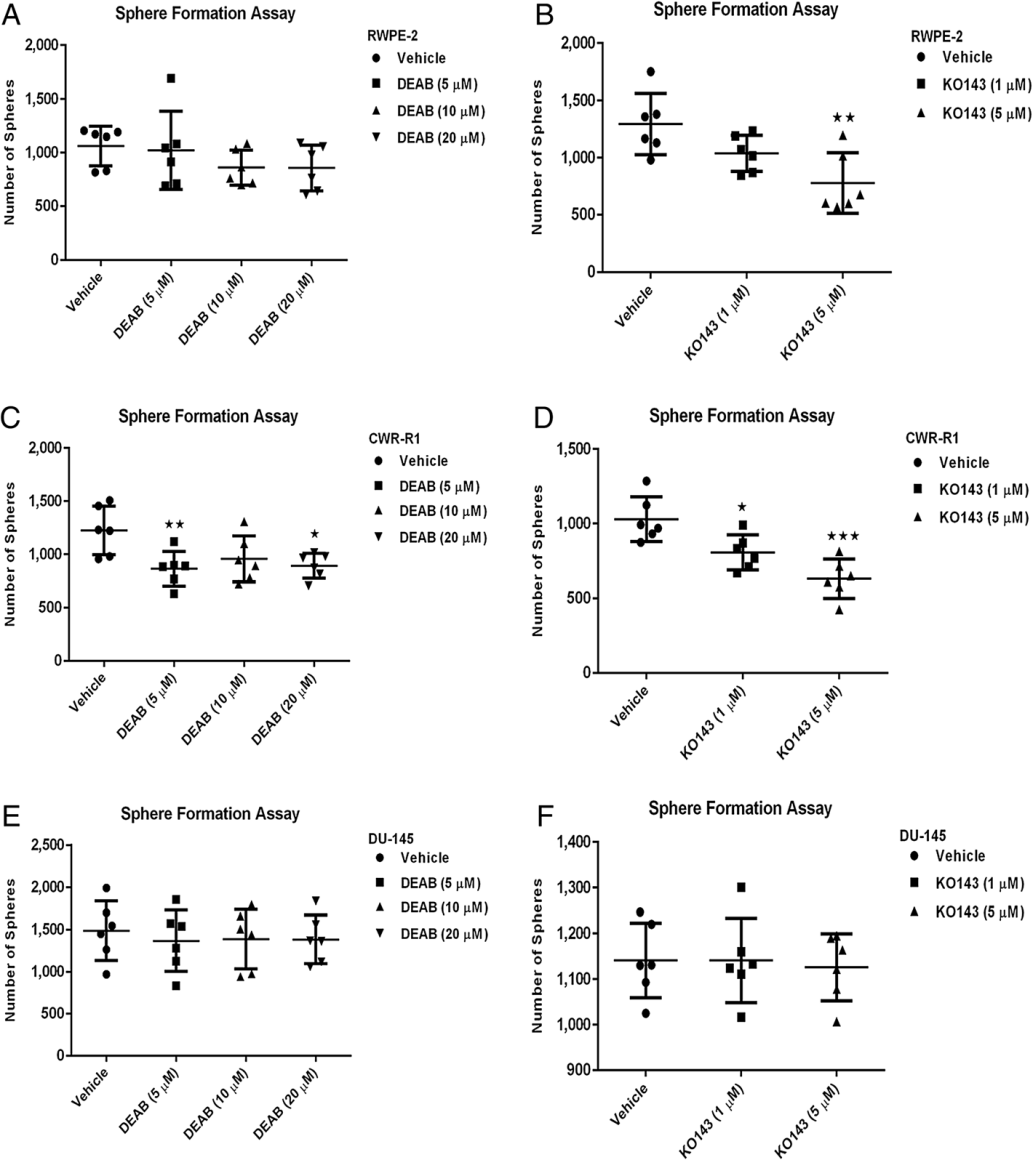
**Sphere formation assay.** RWPE-2, CWR-R1, and DU-145 prostate cells were treated in the presence of DEAB at 5, 10 and 20 μM or Ko143 at 1 and 5 μM concentration or vehicle control. Spheres were counted in each well after 10 to 14 days of growth. Sphere forming ability of RWPE-2 cells did not decrease upon treatment with DEAB **(A)**. Sphere formation was decreased in RWPE-2 cells upon treatment with Ko143 at 5 μM concentration **(B)**. Sphere formation was decreased in CWR-R1 cells upon treatment with DEAB **(C)** or Ko143 **(D)**. Sphere forming ability of DU-145 cells did not decrease upon treatment with DEAB **(E)** or Ko143 **(F)**.

To determine the requirement of ALDH function in stem cell maintenance, sphere formation capabilities were tested in the presence and absence of DEAB, an ALDH1 inhibitor. Sphere formation capability of RWPE-2, CWR-R1 and DU-145 prostate cells was detected upon inhibition of ALDH by DEAB. RWPE-1 cells did not form spheres (data not shown). Though sphere formation was significantly decreased in the CWR-R1 cells upon inhibition of ALDH activity (Figure 5C), sphere formation was not decreased in either RWPE-2 (Figure 5A) or DU-145 (Figure 5E) prostate cells. RWPE-2 had the highest percentage of ALDH^Hi^ cells (Figure 1C) suggesting ALDH activity may not be important in stem cell function.

The side population assay is another functional assay used to isolate stem cells. For comparison, ABCG2 function was inhibited in the prostate cell lines with Ko143. Sphere formation was significantly decreased in both RWPE-2 (Figure 5B) and CWR-R1 cells (Figure 5D) upon inhibition of ABCG2 activity. Sphere formation was not altered in DU-145 cells (Figure 5F) indicating AR may be required for ABCG2 regulation of stem cell maintenance. Taken together ALDH activity represents a marker of prostate stem cells but ALDH activity may not be required for stem cell maintenance.

### Gene expression in cells isolated by stem cell functional assays

**Figure 6:**
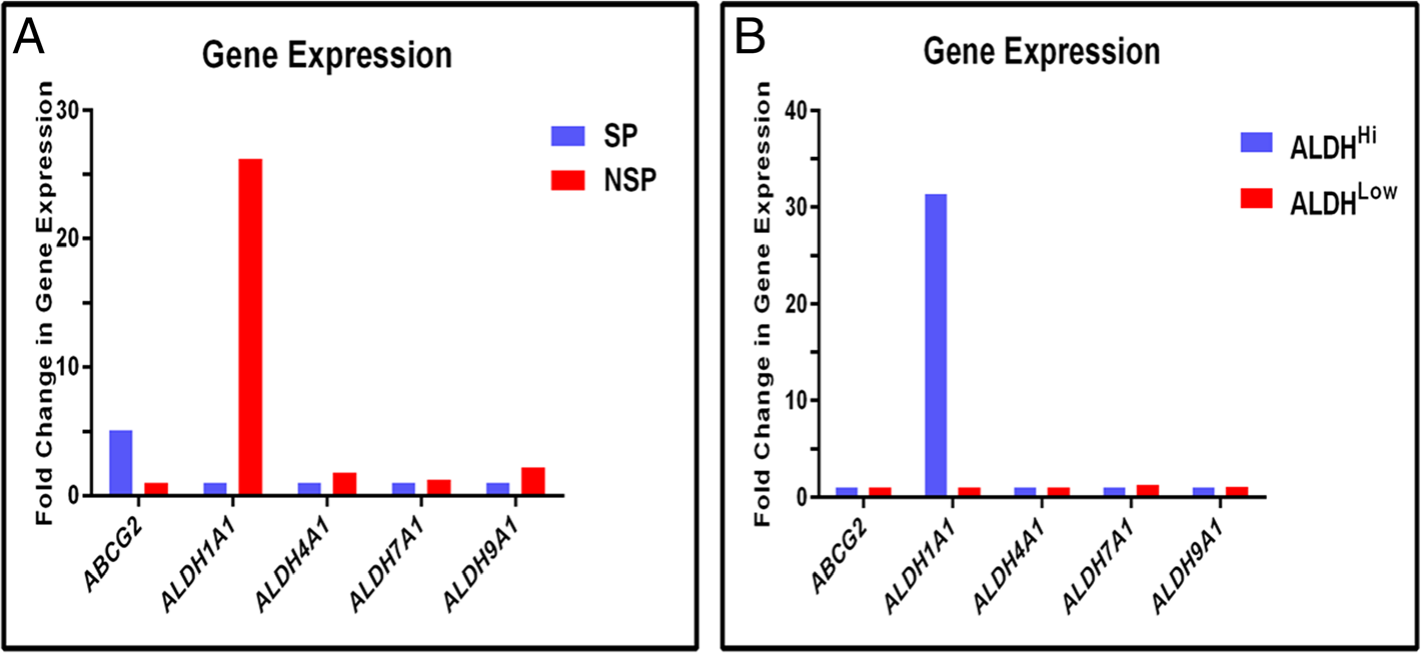
**Gene expression in ALDH**^**Hi**^**, ALDH**^**Low**^**, side population, and non-side population cells isolated from CWR-R1 cells. ***ABCG2*, *ALDH1A1*, *ALDH4A1*, *ALDH7A1* and *ALDH9A1* gene expression was detected by real-time PCR in CWR-R1 cells isolated by the side population and ALDEFLUOR™ assays. Gene expression was normalized to *GAPDH* and fold change in gene expression in each population was calculated based on the formula: 2^-(ΔΔdCt)^. *ABCG2* expression was the highest in the side population with 5.4-fold increase compared to non-side population **(A)**, whereas *ALDH1A1* expression was highest in NSP **(A)** and ALDH^Hi^ cells **(B)**. Approximately 26-fold and 30-fold increase in *ALDH1A1* gene expression was observed in NSP **(A)** and ALDH^Hi^ cells **(B)** respectively as compared to their counter populations. Expression of *ALDH4A1*, *ALDH7A1* and *ALDH9A1* genes was not significantly different in any of the populations **(A** and **B)**. NSP, Non-Side Population; SP, Side Population.

Real-time PCR was performed on CWR-R1 cells isolated by the ALDEFLUOR™ and side population assays to determine the expression of *ABCG2* and specific *ALDH* isoforms. The CWR-R1 cell line was chosen because sphere formation depended on both ALDH and ABCG2 activity. *ABCG2* expression was approximately five-fold higher in the side population compared to the non-side population, thus the side population assay selects for cells with high *ABCG2* expression (Figure 6A). *ALDH1A1* gene expression was approximately 30-fold higher in ALDH^Hi^ cells compared to ALDH^Low^ cells indicating the ALDEFLUOR™ assay enriches for cells with high *ALDH1A1* expression (Figure 6B). The expression of *ALDH4A1, ALDH7A1* and *ALDH9A1* genes was not significantly altered either the side or ALDH populations (Figure 6A, B). Additionally, *ALDH1A1* gene expression was higher in non-side population cells compared to the side population (Figure 6A). Thus the ALDEFLUOR™ assay does not select for cells expressing *ABCG2* and side population assay does not select for cells with expressing *ALDH1A1*.

### Comparison of cells isolated based upon ALDEFLUOR™ and side population assays

**Figure 7:**
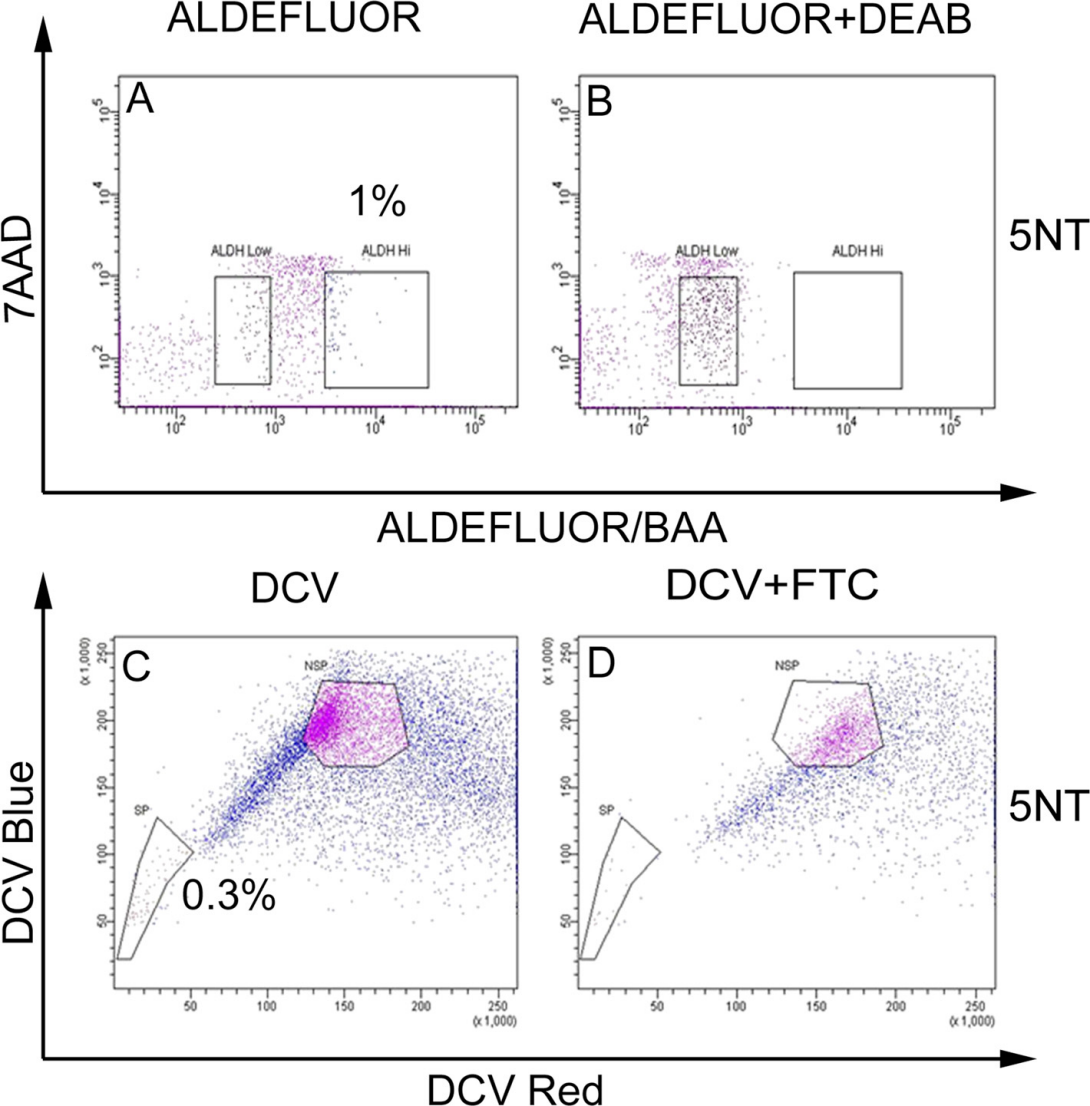
**Isolation of ALDH**^**Hi**^**, ALDH**^**Low**^**, side population and non-side population cells from clinical human prostate specimen.** Cells isolated from clinical human prostate specimen were stained with either DCV or ALDEFLUOR™ reagent. The ALDH^Hi^ cells were detected in the higher fluorescent region **(A)**. ALDH^Hi^ population was inhibited with DEAB resulting in no ALDH^Hi^ cell detection **(B)**. Side population was gated based upon its absence in the presence of FTC, a specific inhibitor of ABCG2 **(C** and **D)**.

To compare stem cell properties of cells sorted based upon the ALDEFLUOR™ or the side population assays, cells were isolated from the same clinical specimens using either assays. Single viable cells from two radical prostatectomy specimens were analyzed with ALDEFLUOR™ and side population assays. The ALDH^Hi^ and ALDH^Low^ cells and side and non-side population cells were isolated from specimens 5NT and 6 T by FACS (Figure 7 and Table 1) and used in tissue recombination.

Recombinants with as low as 50 side population cells could serially generate human prostate tissue for two generations (Table 5). Recombinants with a minimum of 250 ALDH^Hi^ cells isolated from the same clinical specimen were required to generate human prostate tissue when serially recombined for two generations (Table 5). Third generation growth was not detected in either population (data not shown). The difference in the efficiencies of prostatic ductal growth in recombinants with ALDH^Hi^ and side population cells is intriguing but needs to be further investigated to determine if one assay is more efficient at enriching stem cells compared to the other.

**Table 5:** **Incidence of ductal growth in ALDH**^**Hi**^**, ALDH**^**Low**^**, side population and non-side population recombinants**

**Prostate Ductal Growth**^**(#Analyzed)**^**[weeks grafted]**									
			**1**^**st **^**generation**				**2**^**nd **^**generation**		
**Specimen**	**# of Cells**	**ALDH**^**Hi**^	**ALDH**^**Low**^	**SP**	**NSP**	**ALDH**^**Hi**^	**ALDH**^**Low**^	**SP**	**NSP**
5NT	50	0^(3)^ [12]	0^(3)^ [12]	2^(3)^ [12]	2^(3)^ [12]			1^(2)^ [15]	2^(2)^ [15]
5NT	250	1^(2)^ [12]	1^(2)^ [12]			1^(1)^ [12]	1^(1)^ [12]		
6 T	125			0^(2)^ [8]	1^(3)^ [8]				0^(1)^ [6]
6 T	250	1^(2)^ [8]	0^(3)^ [8]	0^(2)^ [8]	0^(3)^ [8]	0^(1)^ [6]			
6 T	500	2^(2)^ [8]	0^(3)^ [8]						
**Total**		**4**^**(9)**^	**1**^**(11)**^	**2**^**(7)**^	**3**^**(9)**^	**1**^**(2)**^	**1**^**(1)**^	**1**^**(2)**^	**2**^**(3)**^

NT, Histologically non-tumor areas; T, Histologically tumor areas from radical prostatectomies; SP, Side Population; NSP, Non-Side Population.

## Discussion

In the current study, we have analyzed the stem cell properties of cells with high ALDH activity with the ALDEFLUOR™ assay and the role of ALDH in stem cell maintenance. Additionally, we have compared the stem cell properties of cells isolated with the ALDEFLUOR™ assay to cells isolated based upon ABCG2 activity, with the side population assay. The requirement for ALDH and ABCG2 activity for stem cell properties was evaluated in tumorigenic prostate cell lines by determining sphere forming capability when ALDH is inhibited by DEAB or ABCG2 is inhibited by Ko143.

As few as 250 ALDH^Hi^ cells from freshly digested and sorted clinical human prostate specimens recombined with rUGM generated prostate tissue for three generations. The serial prostate regenerative capability of ALDH^Hi^ cells increased when higher numbers (500 to 2,000 ALDH^Hi^ cells) were used in tissue recombination assays. Similarly, studies using breast and prostate cancer cell lines showed low tumor growth frequency using 500 ALDH^Hi^ cells, and tumor growth frequency was increased when higher numbers (5 × 10^4 to^ 1 × 10^5^) of ALDH^Hi^ cells were used [8,18,30,31]. Furthermore, only tumors derived from ALDH^Hi^ PC-3 cells are composed of both ALDH^Hi^ and ALDH^Low^ cells demonstrating differentiation capability [18]. The percentage of ALDH^Hi^ cells obtained in our studies in RWPE-1, RWPE-2, CWR-R1 and DU-145 prostate cell lines ranged from 0.5 to 6%. These ranges are consistent with previous studies with PC-3 and LNCaP prostate cell lines [18]. We identified side populations in RWPE-2 (0.4%) (Additional file 3: Figure S3), and previously we have identified the side population in RWPE-1 (0.04%), CWR-R1 (0.4%) and DU-145 (0.73%) cell lines [22]. The percentage of cells in the side population is lower in most cell lines, except in the androgen insensitive DU-145 cell line. Thus, the side population appears to be more selective compared to the ALDEFLUOR™ assay in prostate cell lines.

Real-time PCR analysis indicates that *ABCG2* expressing cells are not enriched in the ALDH^Hi^ population, and *ALDH1A1* expression is not enriched in the side population (Figure 6A, B). Thus, the cells isolated with the two assays were different populations. Both functional assays were not performed simultaneously because the assays are not compatible [2]. This is in contrast to the hematopoietic system, wherein quiescent CD34^+^CD38^Low^ side population cells were highest in the ALDH^Hi^ population, and the combination of assays produces the most enriched primitive hematopoietic stem/progenitor cell population [32].

The sphere formation assay has been shown as an *in vitro* correlate to test for the presence of stem cells [33]. ALDH^Hi^ cells from ovarian, breast and prostate cell lines have more sphere-forming and tumorigenicity capability compared to ALDH^Low^ cells [18-20,30,31,33,34]. Sphere formation decreased in CWR-R1 cells upon treatment with DEAB, suggesting that ALDH activity is important for maintaining stem cell properties in CWR-R1 prostate cells. Sphere-forming ability of RWPE-2 and DU-145 prostate cells did not decrease upon treatment with DEAB. Concurrently, we tested the requirement of ABCG2 activity for sphere formation using Ko143, an ABCG2 specific inhibitor. ABCG2 activity is required for the formation of spheres in RWPE-2 and CWR-R1 prostate cells. However, the sphere-forming ability of androgen insensitive DU-145 prostate cells did not decrease upon treatment with Ko143, suggesting the requirement of androgen signaling for ABCG2 dependent stem cell properties. ABCG2 mediated androgen efflux regulates nuclear AR expression [35]. Thus, ABCG2 activity in AR deficient cells may not be required for stem cell properties.

Recombinants with ALDH^Hi^ cells had human ductal growth in the first generation at a high frequency, and each recombinant contained a high number of glands compared to the recombinants generated with ALDH^Low^ cells. Results from IHC analysis showed that ALDH^Hi^ recombinants contained ALDH1A1 expressing cells, thus demonstrating self-renewal capabilities, nuclear AR and the basal cell marker p63. Additionally, ALDH1A1 expression was also detected in non-basal cells in the recombinants as illustrated by cytoplasmic staining. An ALDH^Hi^ recombinant showed that ALDH1A1 positive glands also expressed ABCG2, PSA and chromogranin A. These results suggest that ALDH1A1 is expressed in heterogeneous, cells with multipotency, some of which possess stem cells properties. As further evidence of differentiation potential, a higher percent of ALDH^Hi^ recombinants with human ductal growth expressed ALDH1A1, ABCG2 and PSA with 19%, 45% and 33% recombinants showing expression, respectively, compared to 2%, 29% and 15%, respectively, in ALDH^Low^ recombinants (Table 4).

None of the recombinants suspended in collagen alone grew prostatic tissue and recombinants with rUGM did not demonstrate prostate cancer pathology. This is not surprising since the presence of an inductive microenvironment, provided by the rUGM in these experiments, has been shown to be critical to trigger prostate generation [36,37]. Previous studies have shown that the presence of inductive mesenchyme can normalize transformed epithelial cells giving rise to normal prostatic growth even from cells isolated from tumor specimens [38-40]. Alternatively, prostate cancer pathology was observed in the tissue recombination assay with rUGM when a high number of prostate cancer cells were used [37]. There could be several possibilities for why no cancer pathology was found in the recombinants using cells isolated based on either the ALDEFLUOR™ or side population assays. Several studies used prostate cancer cell lines, increasing the possibility of isolating cancer stem cells. The number of cells used in this study, 50 to 2,000, may not be enough to implant the rare cancer stem cell. There could also be patient variability with respect to the presence of cancer stem cell markers accounting for the presence of fewer side populations or ALDH^Hi^ cells in different specimens. Alternatively, there could be cancer stem cells expressing different markers, other than ABCG2 or ALDH activity, that have the capability to initiate prostate tumor growth. Prostate cancer stem cells from human specimens capable of forming prostate tumor pathology in the recombination assay were not isolated by the side population or ALDEFLUOR™ assays. Nevertheless, results from the tissue recombination assay showed that there is serial transplantable and prostate regenerative capability in recombinants with side population and ALDH^Hi^ cells.

Previous reports from our laboratory have identified side population cells in different prostate cell lines and freshly digested human prostate specimens [22,26]. Furthermore, side population cells isolated from human prostate specimens generated prostatic tissue for multiple generations in the tissue recombination assay [26]. Therefore, we wanted to directly compare putative prostate stem cells isolated based upon ABCG2 or ALDH activity. Since, the ALDH product formed in the ALDEFLUOR™ reaction is effluxed by the ABCG2 transporter [2], the side population and the ALDEFLUOR™ assays were performed separately. The specimens contained 1% ALDH^Hi^ cells and about 0.3% of the cells in the specimens demonstrated the side population phenotype. Results from the tissue recombination assay with side population cells are consistent with our previous studies [26]. In our current study, we have observed serial generation of prostate growth with as low as 250 ALDH^Hi^ and 50 side population cells. While recombinants with as low as 50 side population cells generated prostate for two generations, a minimum of 250 ALDH^Hi^ cells were required to serially generate prostate for two generations. Results from the tissue recombinant assay comparing the prostate regeneration capability of side population and ALDH^Hi^ cells suggested that a higher percentage of cells with ABCG2 activity have stem cell potential compared to cells with high ALDH activity.

## Conclusions

This is the first study to demonstrate that ALDH^Hi^ cells isolated from human prostate tissue demonstrate prostate tissue growth in the tissue recombination assay. Cells isolated based on ABCG2 activity have a higher frequency of stem cell potential compared to ALDH^Hi^ cells and the ALDEFLUOR™ assay does not enrich for *ABCG2* expressing cells. The ALDEFLUOR™ assay does isolate prostate stem cells, but the side population assay enriches for prostate cells with more stem cell potential.

## Abbreviations

7-AAD: 7-Amino actinomycin D; ABCG2: ATP binding cassette transporter G2; ALDH: Aldehyde dehydrogenase; ALDH1A1: Aldehyde dehydrogenase1A1; ALDHHi: High aldehyde dehydrogenase1A1 activity isolated based upon ALDEFLUOR™ assay; ALDHLow: Low aldehyde dehydrogenase1A1 activity isolated based upon ALDEFLUOR™ assay; AMACR: Alpha-MethylAcyl-CoA Racemace; AR: Androgen receptor; DCV: Dye cycle violet; DEAB: Diethyl amino benzaldehyde; FACS: Fluorescence activated cell sorting; FISH: Fluorescence *in situ* hybridization; IHC: Immunohistochemistry; PCR: Polymerase chain reaction; PSA: Prostate specific antigen; RPCI: Roswell park cancer institute; rUGM: Rat urogenital mesenchyme.

## Competing interests

The authors have read the journal’s policy and have the following conflicts: WJH is a RPCI patent holder for “Methods for evaluating and implementing prostate disease treatments” Patent No. 8048640. All other authors do not have any conflicting interests.

## Authors’ contributions

KJG, BAF and WJH conceived and designed the experiments. KJG, BAF and WJH performed the experiments and analyzed the results. GA and CDM performed pathology analysis. AM performed statistical analysis. CDM contributed the reagents/materials/analysis tools. KJG, BAF and WJH wrote the paper. All authors read and approved the final manuscript.

## Supplementary Material

Additional file 1: Figure S1 Positive and negative controls for telomere FISH analysis. Mouse control tissue **A**. Telomere FISH analysis positive for telomere repeats; **B**. DAPI counter stain same field; Human control tissue **C**. Telomere FISH analysis negative for telomere repeats; **D**. DAPI counter stain same field. Scale bar = 10 μm. Click here for file

Additional file 2: Figure S2 Positive and negative human prostate controls for IHC staining. **A**. p63 IHC; **B**. no primary antibody (p63) with goat anti-mouse IgG antibody IHC; **C**. AR IHC; **D**. no primary antibody (AR) with goat anti-rabbit IgG antibody IHC; **E**. PSA IHC; **F**. no primary antibody (PSA) with goat anti-mouse IgG antibody IHC; **G**. ABCG2 IHC; **H**. no primary antibody (ABCG2) with goat anti-mouse IgG antibody IHC; **I**. Chromogranin A IHC; **J**. no primary antibody (Chromogranin A) with goat anti-rabbit IgG antibody IHC. **K**. ALDH1A1 IHC; **L**. no primary antibody (ALDH1A1) with goat-anti-rabbit IgG antibody IHC. Scale bar = 50 μm. Click here for file

Additional file 3: Figure S3 Isolation of side population and non-side population cells from RWPE-2 prostate cells. RWPE-2 prostate cells were stained with DCV reagent. The side population was gated based upon its absence in the presence of FTC, a specific inhibitor of ABCG2 (**A** and **B**). Click here for file
