# Supplementary figures and images for: Aldehyde dehydrogenase and ATP binding cassette transporter G2 (ABCG2) functional assays isolate different populations of prostate stem cells where ABCG2 function selects for cells with increased stem cell activity
Source: Stem Cell Res Ther. 2013 Oct 25;4(5):132. doi: 10.1186/scrt343 (PMC3854760; doi:10.1186/scrt343)

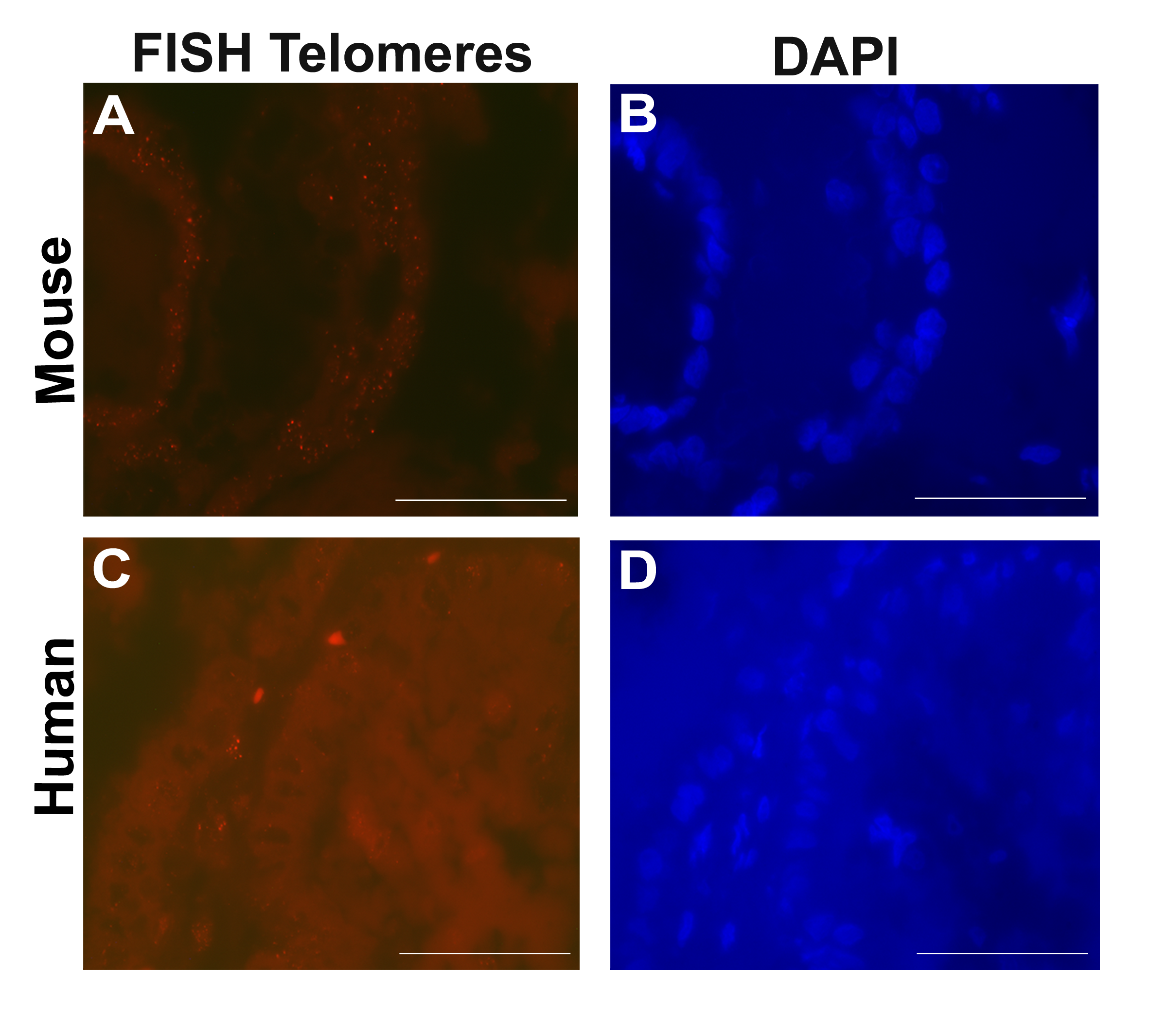

Supplement: Additional file 1: Figure S1 — Positive and negative controls for telomere FISH analysis. Mouse control tissue A. Telomere FISH analysis positive for telomere repeats; B. DAPI counter stain same field; Human control tissue C. Telomere FISH analysis negative for telomere repeats; D. DAPI counter stain same field. Scale bar = 10 μm. [file scrt343-S1.tiff]

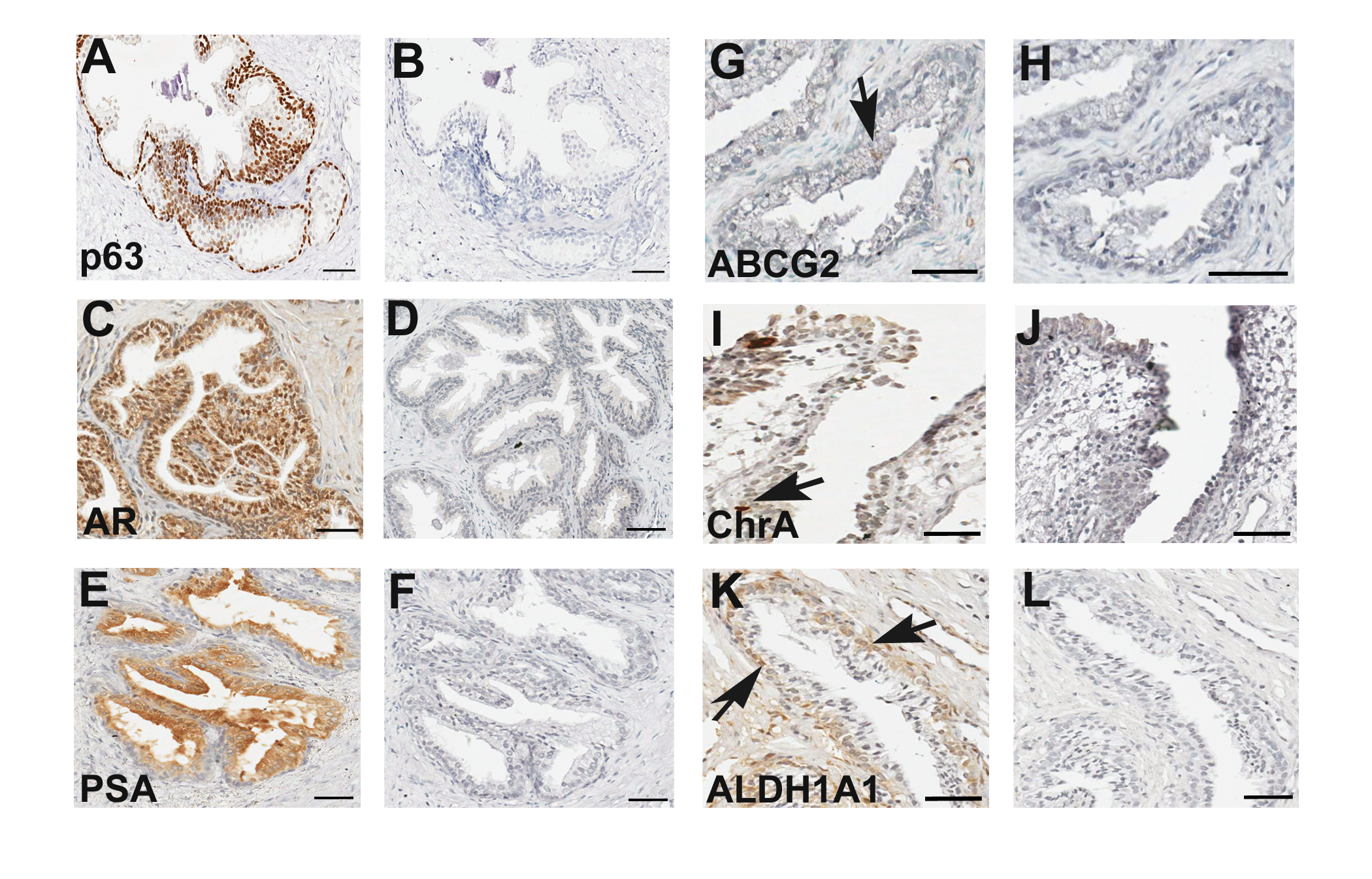

Supplement: Additional file 2: Figure S2 — Positive and negative human prostate controls for IHC staining. A. p63 IHC; B. no primary antibody (p63) with goat anti-mouse IgG antibody IHC; C. AR IHC; D. no primary antibody (AR) with goat anti-rabbit IgG antibody IHC; E. PSA IHC; F. no primary antibody (PSA) with goat anti-mouse IgG antibody IHC; G. ABCG2 IHC; H. no primary antibody (ABCG2) with goat anti-mouse IgG antibody IHC; I. Chromogranin A IHC; J. no primary antibody (Chromogranin A) with goat anti-rabbit IgG antibody IHC. K. ALDH1A1 IHC; L. no primary antibody (ALDH1A1) with goat-anti-rabbit IgG antibody IHC. Scale bar = 50 μm. [file scrt343-S2.tiff]

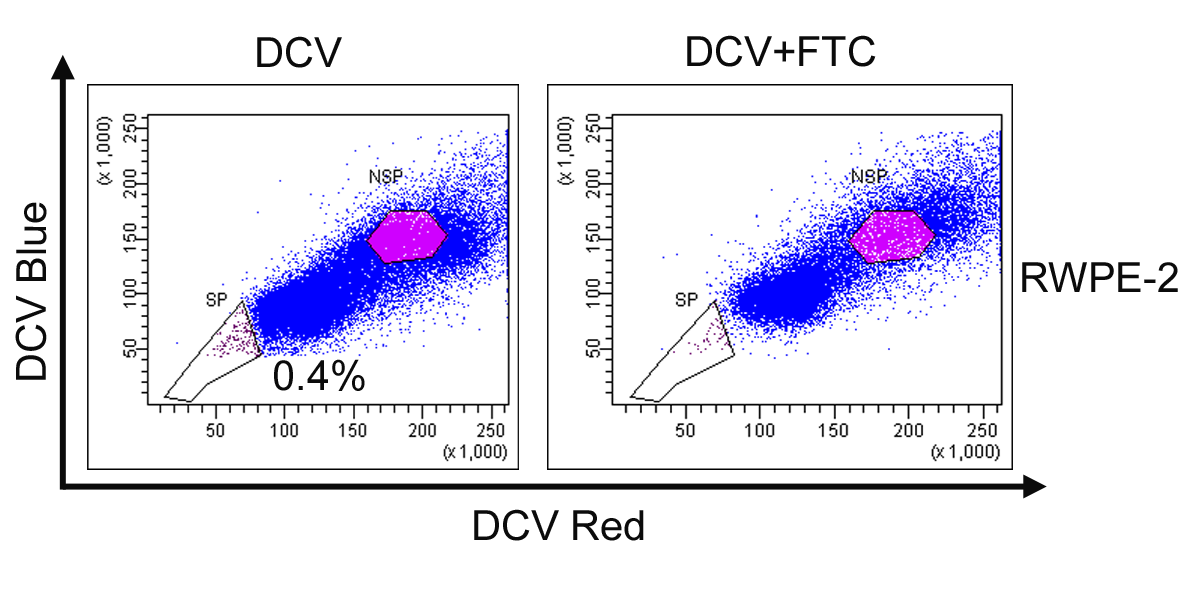

Supplement: Additional file 3: Figure S3 — Isolation of side population and non-side population cells from RWPE-2 prostate cells. RWPE-2 prostate cells were stained with DCV reagent. The side population was gated based upon its absence in the presence of FTC, a specific inhibitor of ABCG2 (A and B). [file scrt343-S3.tiff]
